# What should I use to calculate vehicle EES?

**DOI:** 10.1371/journal.pone.0297940

**Published:** 2024-02-08

**Authors:** Pavlína Moravcová, Kateřina Bucsuházy, Robert Zůvala, Marek Semela, Albert Bradáč

**Affiliations:** 1 Transport Research Centre, Brno, Czech Republic; 2 Institute of Forensic Engineering, Brno University of Technology, Brno, Czech Republic; Jamia Millia Islamia, INDIA

## Abstract

Comprehensive crash analysis includes calculating impact speed, which requires the determination of kinetic energy expended on the deformation of the vehicle’s structural elements at the point of contact during a collision. The accuracy of the input data affects the resulting analysis of the crash. Therefore, this article aims to analyse selected factors influencing the determination of Energy Equivalent Speed (EES) determination using the CRASH3 algorithm: the extent of damage using defined measurement points, deformation width, and also limit speed b_0_. The variables were varied depending on selected factors such as the extent of damage, the type of collision (overlap), and also vehicle type (vehicle category classification). The presented study concluded that using 2 equally spaced measurement points to define the deformation profile should not be recommended in forensic practice when using CRASH3 algorithm. Using 7 measurement points seems more appropriate in case of equal spacing, even though the differences in calculated EES are not high when using 5 or 6 measurement points, especially with respect to the inaccuracy/technically acceptable tolerance of the EES value determination. The resulting EES is significantly influenced by variation of the deformation width. The used b_0_ range had a significant effect on the resulting EES value only in the case of SUVs. These vehicles show higher stiffness, which supposes the use of lower b_0_ values should not be recommended.

## Introduction

High-quality documentation of a crash (crash scene and damaged vehicles) is one of the most important prerequisites for subsequent crash analysis [1]. Crash analysis (in European countries mostly conducted by expert witnesses) can serve as an important basis for determining crash culpability. Incorrect damage extent analysis could result in incorrect determination of the vehicle impact speed, which could lead to an incorrect conclusion regarding crash culpability. Correct crash analysis requires an inverse approach–determination of causation is based on track documentation from the crash scene. The input data accuracy significantly impacts the result [2].

Various methods are used for estimating the relative velocity or delta V of two colliding vehicles at impact by simply evaluating energy loss during the collision [3]. The determination of the deformation energy after crash is based on the measurement of the deformation depth [2]. Measuring deformation depth can in some cases be problematic (with respect to the available data and the character and scope of the vehicle damage). It is also necessary to consider the limits of some measurement methods [4–6].

Deformation energy is expressed by the Equivalent Energy Speed (EES), which represents the amount of energy required for permanent deformation. Kinetic energy is expended on the deformation of the vehicle’s structural elements at the point of contact during a collision. Depending on the crash analysis used, the EES parameter can serve as a control parameter or directly as part of the collision speed calculation, e.g., using the Energy ring or using the Law of Conservation of Momentum or Law of Conservation of Energy [7–10]. In the field of crash reconstruction, the Calspan Reconstruction of Accident Speeds on the Highway, version 3 –CRASH3 calculation algorithm is often used for determining the deformation energy including quantification of the EES [11, 12].

### CRASH3

The CRASH3 damage algorithm was established initially in the 1970s for estimating the deformation energy of light utility vehicles (LUV) [11, 13] and is based on Campbell´s method [12, 14–16]. The algorithm has been subject to numerous papers and included in several software programs for crash reconstruction for decades [12, 15, 16].

Several papers compared the accuracy of the CRASH3 algorithm with the results of crash tests [11]. Rose and Carter [17] pointed out the systematic error of deformation depth measurement which occurs when using crash tests. The error arises because of the plastic bumper fascia, which rebounds more than the underlying structure. One of the limitations of the algorithm is the assumption of the linear dependence of the impact speed and force on the deformation depth, which could affect the deformation energy calculation (respectively EES parameter) [18]. Among other factors, the assumption of homogeneous stiffness across the entire vehicle front part can also significantly affect the calculation results. The EES value of a crash with 20% overlap will be overestimated as the greater stiffness of the vehicle parts will be used for the calculation. In contrast, during a collision with a narrow obstacle the resulting EES will often be underestimated due to the consideration of lower stiffness [18].

Some previous studies aimed to determine the influence of selected factors such as the deformation depth area, deformation depth measurement, or improvement of the methodology using a higher number of measurement points on the resulting deformation energy or measurement of deformation depth [11, 19, 20]. Wood et al. [21] analysed the relationship between stiffness coefficients and variables such as wheelbase, model year, front overhang or front axle weight. Even though vehicle wheelbase has not changed significantly over the years, stiffness coefficients have changed due to individual factors, such as structural and technological advances. Some of the studied factors such as wheelbase are not necessarily sufficient to predict the stiffness coefficients, but combining them increases the probability of a more accurate prediction of the stiffness coefficient [21]. Singh [22] analysed the vehicle-dependent nature of changes in stiffness coefficients with increasing levels of mesh fineness. The appropriate mesh fineness selection is based on the specificity of the crush profiles and mainly the reconstructing engineer’s judgment.

### EES determination using CRASH3 in PC-Crash software

For determination of the EES it is necessary to determine stiffness coefficient A, B and G (G is calculated from A and B as equal to A^2^/2B). These stiffness coefficients are individual for each vehicle and impact area [2]. The deformation profile–deformation width and depth at selected measurement points and limit speed with no visible deformation on vehicle b_0,_ also need to be defined.

Limit speed b_0_ is the maximum flat fixed barrier impact speed which produces no residual crushing. Previous papers refer to several b_0_ value ranges [2, 11, 14, 17, 23–25] in relation to the type of impact (front, rear or side impact), the wheelbase, vehicle type, etc. Used b_0_ values usually range from 6 km/h up to 14 km/h. Rose and Carter [17] and Brach et al. [11] use a range from 4 to 7 mph (6.3 to 11.2 km/h). In Daily et al. [14] a value of 5 mph (8 km/h) is typically assigned for front and rear impacts and a value of 2 mph (3 km/h) for side impacts. Vangi [2] and Gaffney et al. [23] state that b_0_ is around 8 km/h for both front and rear impacts and basically constant for all vehicles. Kubiak et al. [24] stated that based on NHTSA data, b_0_ ranges from 2 to 4 m/s (7.2 to 14.4 km/h). Osterholt et al. [25] calculated b_0_ for different vehicle categories split up also by wheelbase.

The measurement of vehicle damage width includes both direct and induced damage [26]. Authors in the past [2, 20, 26, 27] also used different approaches to measuring the deformation width. The width of deformation used to be measured between marginal damage points the or the entire width of front part. In case of partial offset crashes, the deformation is often measured from the margin of the deformation area to the edge of the vehicle (see e.g. [2, 20, 26, 27]). As described by Nordhagen et al. [27], various models are used because not all are universally applicable and rely on the crash circumstances and methodology. It is important that the crush measurement methodology match the model used.

The deformation profile is determined using several measurement points to determine the extent of the vehicle damage from its original profile. Crush profile is a two-dimensional representation of the deformation, so the representative 2D damage profile needs to be selected at the elevation where the more significant load-bearing structure is located. The deformation depth measurement is based on the Tumbas and Smith Measuring Protocol for Quantifying Vehicle Damage from an Energy Point of View [26]. The measurement is perpendicular to the plane of the damaged side and measurements are equally spaced across the damage width. [11, 26, 28, 29]. In front and rear crashes, crush should be measured at the height of the vehicle frame to ensure that the measurement is associated with the major force transfer in the impact. In side impacts, crush should be measured at the level of maximum deformation for side impacts [28, 30]. The “original” CRASH3 algorithm which is based on guidelines by Tumbas and Smith [28] recommend using two, four or six measurement points of the deformation profile. As described by Cookson [30], the measurement needs to be carried out at a minimum of three equally spaced points. The equally spaced measurement profile and limited number of measurement points may not adequately reflect the actual deformation profile [31]. As shown in the example of one vehicle crush energy calculation, using only 3 measurement points leads to a 14% change in the resulting calculated crush energy, while using seven measurement points resulted in only a 0.63% reduction. Seven well-chosen points appear to suffice, even for a severe side impact [31]. Vangi [2] states that even irregular deformation profiles can be linearized by approximating the damaged area with triangular, rectangular or trapezoidal geometries which could simplify the Deformation Energy calculation. The traditionally used formulas for stiffness coefficient calculation (also used by Pc-Crash software) require equally spaced crush measurements [31].

### Introduction summary

To calculate EES it is necessary to define various variables, among others to determine the damage extent using defined measurement points. Variables used for the EES calculation are used in a varying range:

- Based on the widely used methodology the deformation depth can be measured at 2–12 measurement points. Usually when using the CRASH3 algorithm in forensic practise, 6 measurement points for determining the deformation profile are widely used, but there are clues that this could be inappropriate.
- An important characteristic affecting the EES calculation is vehicle stiffness. Elastic stiffness is expressed, among others, by the b_0_ parameter, which varies depending on the type of impact and vehicle, and usually ranges from 6 km/h up to 14 km/h.
- The measurement methodology for the damage width also varies–in full-overlap impact the full vehicle width or bumper width is used, similarly in the case of damage offset, where the width is one side bounded by deformation.

Various papers show differences in quantified crush energy in relation to different methodologies and the used variables range but primarily only using representative examples without more detailed analysis. The paper aims to analyse the influence of different commonly used measurement methodologies and selected variables’ value range on the resulting EES value.

## Methods

The selected variables–namely deformation width L_d_, limit speed b_0_, and a number of measurement points–were varied and the influence on the resulting EES value using the CRASH3 calculation algorithm was analysed. Parameters such as vehicle type and extent of deformation will also be considered.

### Data set

For measurement and subsequent analysis, the EES values of 20 vehicles with different stiffness characteristics were calculated, and the selected parameters were varied. All selected vehicles were damaged in the frontal part, but the extent of deformation differs (full-overlap, damage off-set, vehicle center damage after crashing into fixed obstacle). The dataset included vehicles of different classes and ages (model years 2000 to 2019). Vehicles were divided by vehicle category classification–small vehicles, smaller medium-sized vehicles and medium-sized vehicles, and SUVs (see Table 1).

**Table 1:** Vehicle characteristics.

vehicle category classification	vehicle	manufacture year	mass [kg]	overlap [%]	deformation extent
**small**	Skoda Fabia	2001	1015	40	damage off-set
	Suzuki Swift	2002	967	100	vehicle center damage
	Renault Clio	2000	945	100	vehicle center damage
	Hyundai Getz	2004	1247	40	full-overlap
	Hyundai Getz	2006	1175	80	damage off-set
**smaller medium-sized**	VW Bora	2000	1555	80	full-overlap
	Skoda Octavia	2004	1460	80	full-overlap
	VW Caddy	2020	1720	80	full-overlap
	Skoda Octavia	2000	1237	100	full-overlap
	Ford Focus	2001	1153	100	full-overlap
**medium-sized**	Skoda Superb	2013	1951	40	full-overlap
	Volvo V50	2007	1542	25	full-overlap
	Audi A4	2004	1480	80	full-overlap
	Skoda Superb	2007	1505	25	full-overlap
	Skoda Superb	2017	1541	50	damage off-set
**SUV**	Skoda Karoq	2019	1483	30	damage off-set
	Skoda Karoq	2019	1603	100	damage off-set
	Skoda Karoq	2019	1448	100	full-overlap
	Skoda Kodiaq	2019	1879	60	damage off-set
	Skoda Karoq	2018	1661	100	full-overlap

### Vehicle deformation documentation and analysis

**Fig 1:**
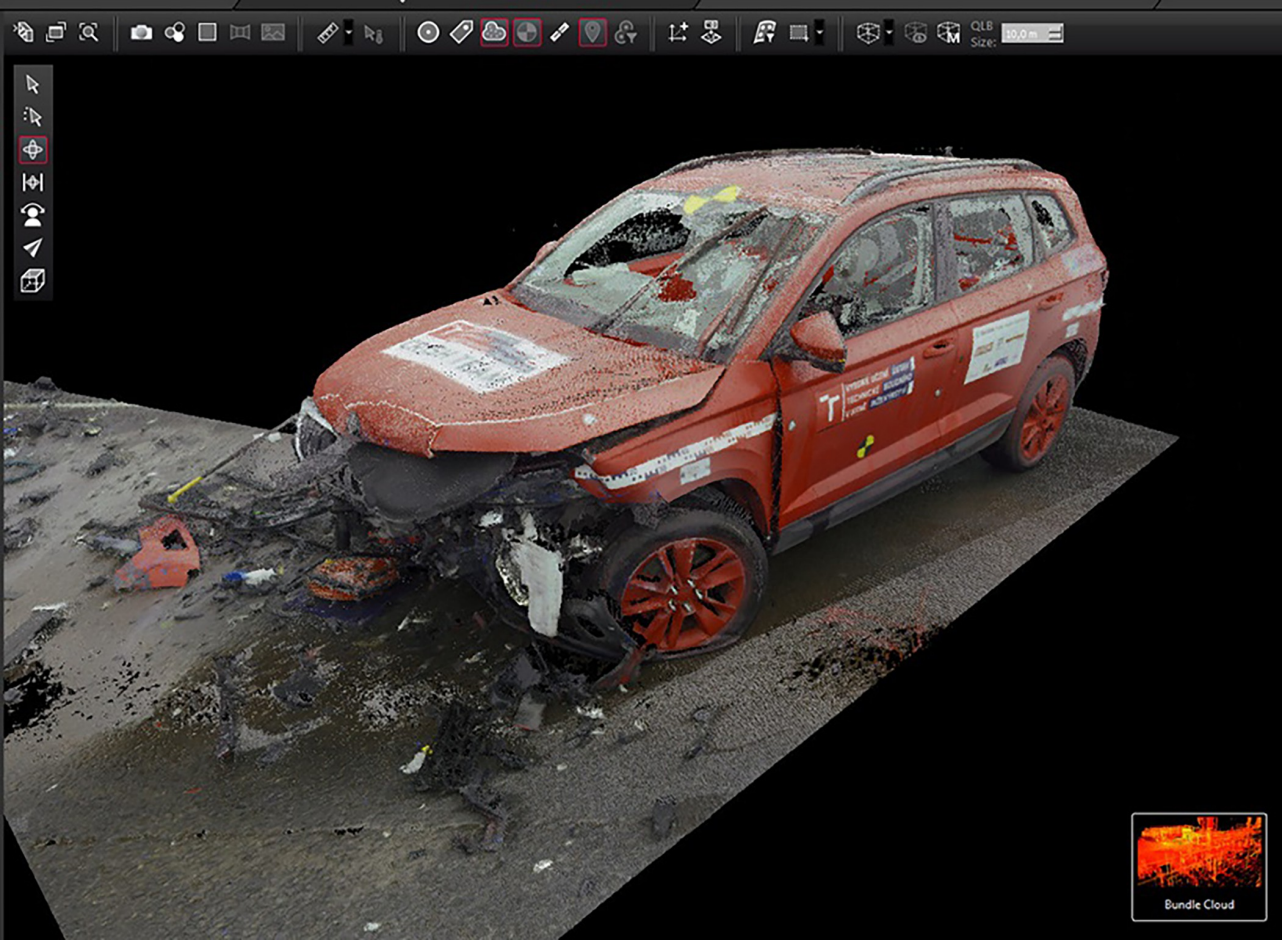
Vehicle deformation documentation.

Vehicle deformation was documented using a 3D laser scanner (Faro Focus 120 laser scanner or a Leica RTC360 –Fig 1) as one of the most precise methods for the documentation [32, 33]. The subsequent analysis was carried out using the Geomagic Control program, where a 2D cut was made at the height of the vehicle impact bar to ensure that the measurement is associated with the major force transfer in the impact. The cut was further converted to.dxf format. The deformation depth was determined based on a comparison with an undamaged 2D model of the vehicle from the AutoView model database software.

### EES calculation

The EES was determined using the CRASH3 algorithm. The selected parameters were varied:

- The number of measurement points in the range C_2_ –C_12_. In the specified zones it is possible to enter the plastic deformation depth C_1_ to C_12_ with a transverse distribution of L_1_ to L_12_. Equal spacing was used (as required by CRASH3 manual–see Tsongos [34].
- The measurement width: in case of full overlap as full vehicle width or bumper width, similarly in case of damage offset, where the width is one side bounded by deformation.
- The b_0_ parameter from 6 up to 12 km/h.

**Fig 2:**
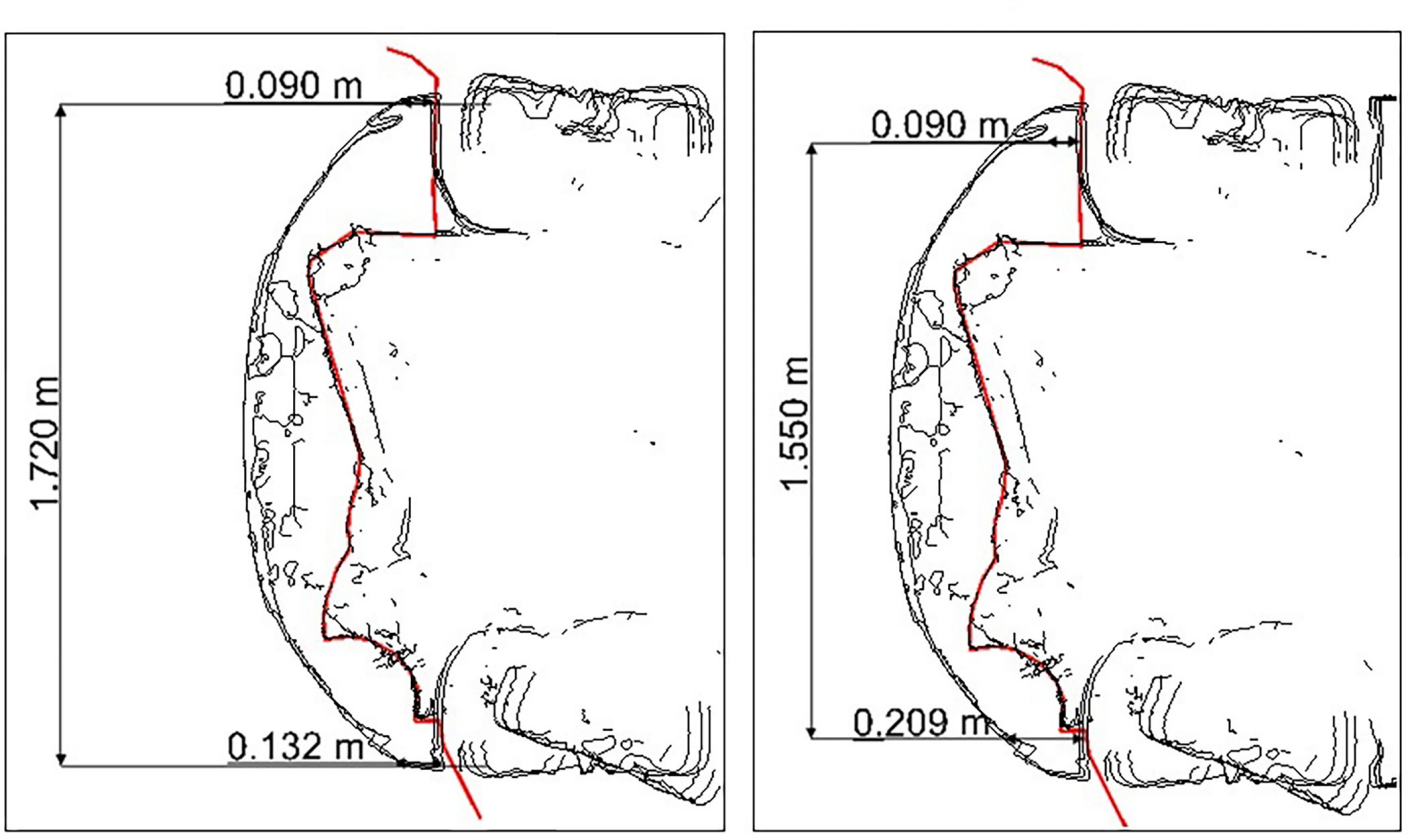
The measurement width: a) vehicle, b) bumper.

**Fig 3:**
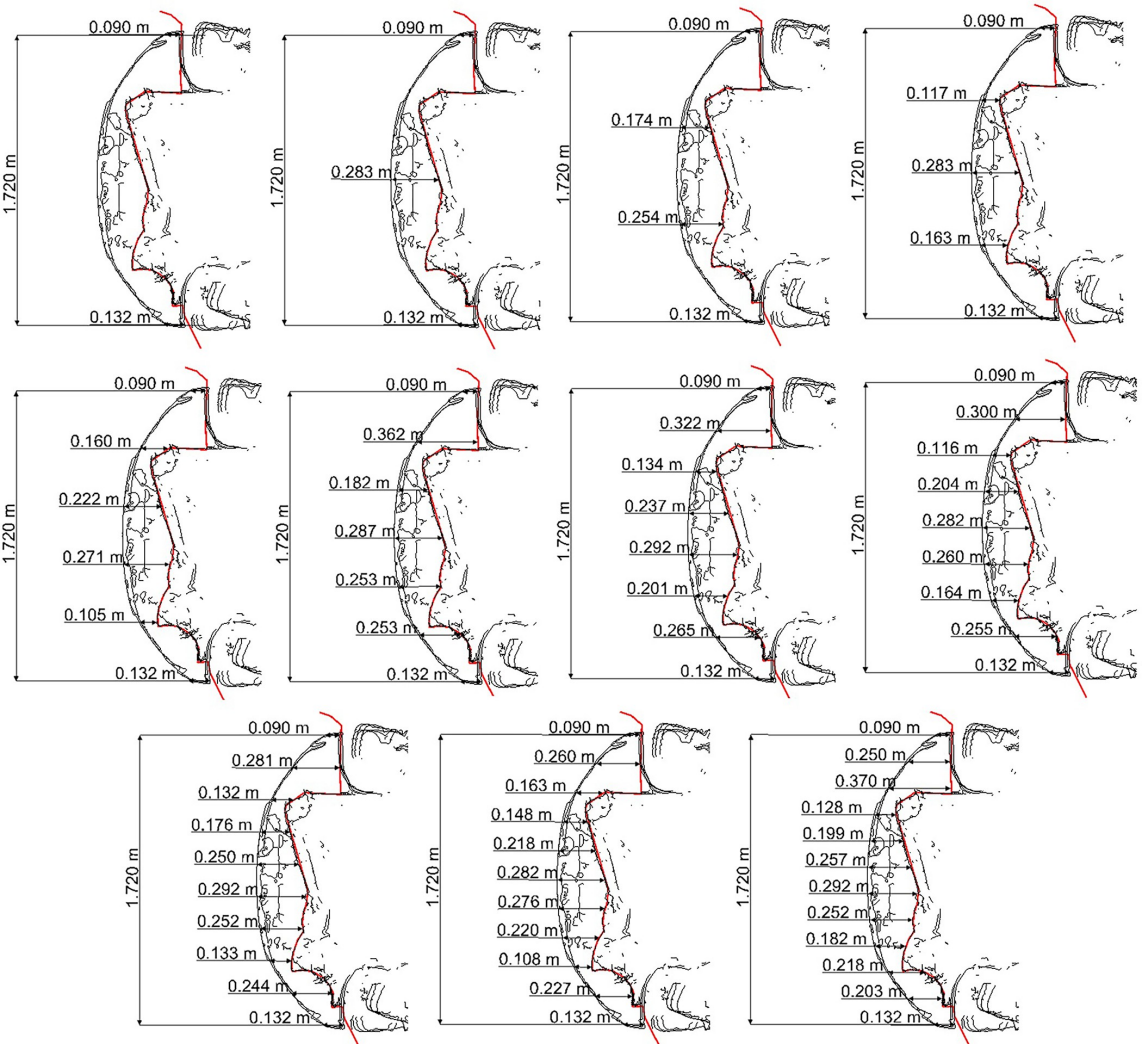
The number of measurement points in the range C2 –C12.

The measurement procedure is illustrated in the following figures (see Figs 2 and 3).

## Results

The paper’s objective was to define the influence of the selected variables on the EES calculation. The resulting EES values were tested with respect to these variables and also with respect to the vehicle type and extent of damage.

The statistical testing considered:

- Vehicle category classification (small vehicle, medium-sized and lower medium-sized, SUVs)
- Damage extent (full overlap, vehicle center, damage offset)
- Deformation overlap

**Fig 4:**
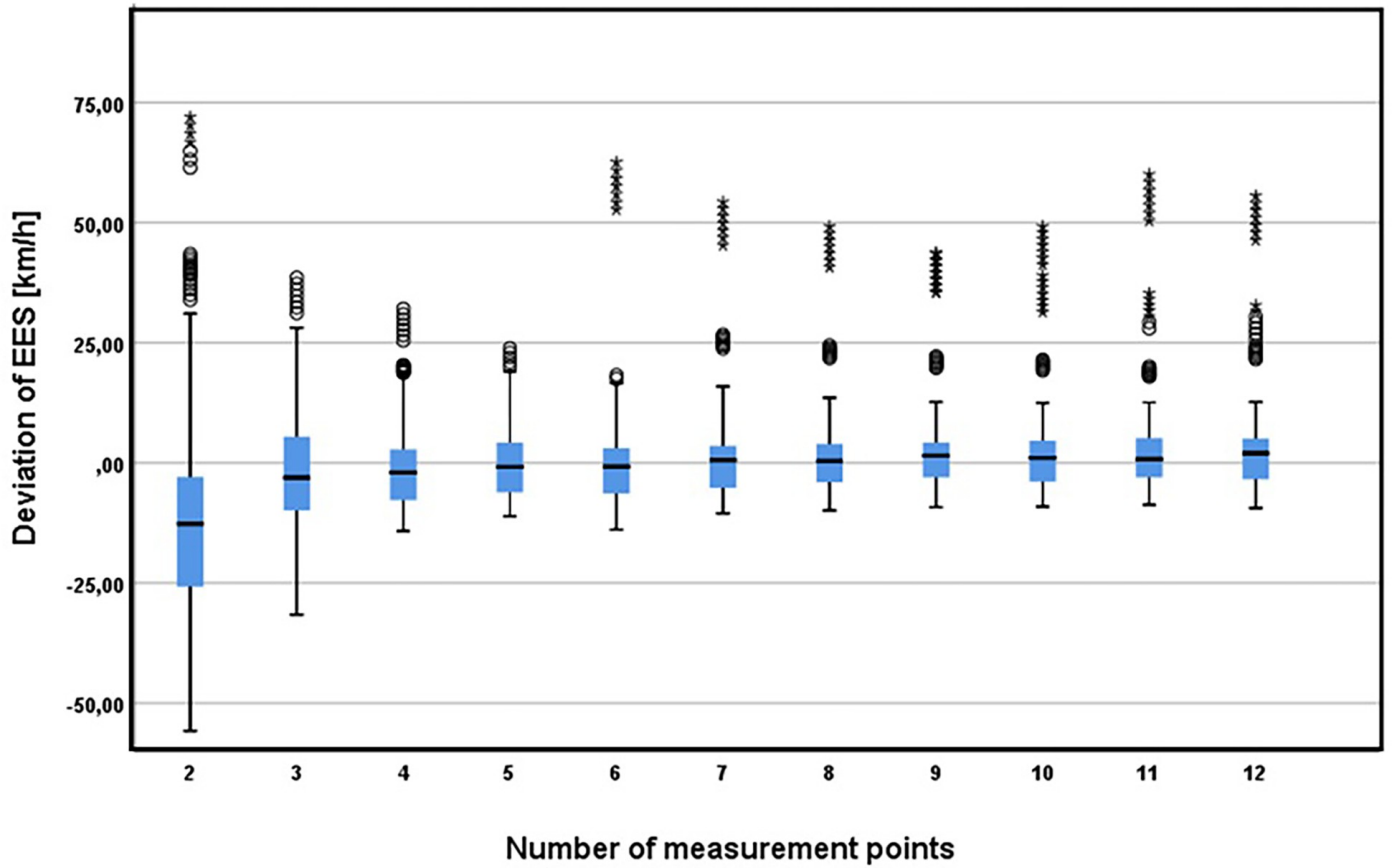
Effect of number of measurement points on the resulting EES values.

The number of measurement points ANOVA indicates statistically significant differences in the resulting EES values using a different number of measurement points C_2_-C_12_ (p value <0.001). The groupwise comparison shows statistically significant differences between EES in relation to 2–6 measurement points. The accuracy of the EES calculation in case of equal spacing increases with the increasing number of measurement points, i.e. higher accuracy of the focused deformation—a higher number of measurement points usually better reflect the shape of the damage. The obtained results are more consistent when more than 6 measurement points are used, as illustrated in Fig 4. EES calculation when using 7, and more measurement points do not differ significantly compared to higher measurement points, so using 7 measurement points seems appropriate in case of equal spacing.

The most significant differences are apparent in case of 2 equally spaced measurement points only. The use of 2 equally spaced points thus appears to be unusable for practical use when using CRASH3 algorithm (considering deformation width from edges of the vehicle). In the case of using only 2 measurement points, instead of an even distribution, it is necessary to choose a distribution that better reflects the character and extent of the deformation. Due to significant deviations when using 2 measurement points, a different deformation measurement methodology was also used–see [22]. The resulting EES values are comparable with a calculation based on a higher number of measurement points. The results are not significantly different in comparison with 7–12 equally spaced measurement points.

- Vehicle category classification: within vehicle classes, the number of measurement points has a statistically significant effect on the resulting EES value. The results reflect the previous findings, that in case of equal spacing, 6 measurement points should be used as a minimum. A lower number of measurement points leads to statistically significant differences in the resulting EES value in all vehicle classes.
- Overlap: the number of measurement points has a significant effect on the resulting EES value. However, even in the case of the analysed small overlaps (dataset contains 25–30% overlaps), it should not be recommended to reduce the number of measurement points. It should be noted that in the analysed scenarios, considerable induced deformation occurred in the case of collisions with an overlap of 25–30%, so the resulting extent of damage was higher.
- Extent of damage: considering the extent of damage, there are statistically significant differences in the determined EES value with respect to the number of measurement points.

The results are least consistent for vehicle centre damage (which in this case means a collision with a fixed obstacle) which reflect the assumption of less usability of CRASH3 for this type of crash. The highest inaccuracy of the resulting EES calculation is associated with 2 equally spaced measurement points, regardless of extent of damage (respectively for all damage types) with an even distribution of measurement points. The higher span in case of two measurement points and full overlap crashes may be influenced by inaccuracy related to crush measurement not considering vehicle front part curvature and hence measuring significantly low crush depth. (edge points can reach almost zero values of crush depth and may not reflect the actual deformation profile).

**Fig 5:**
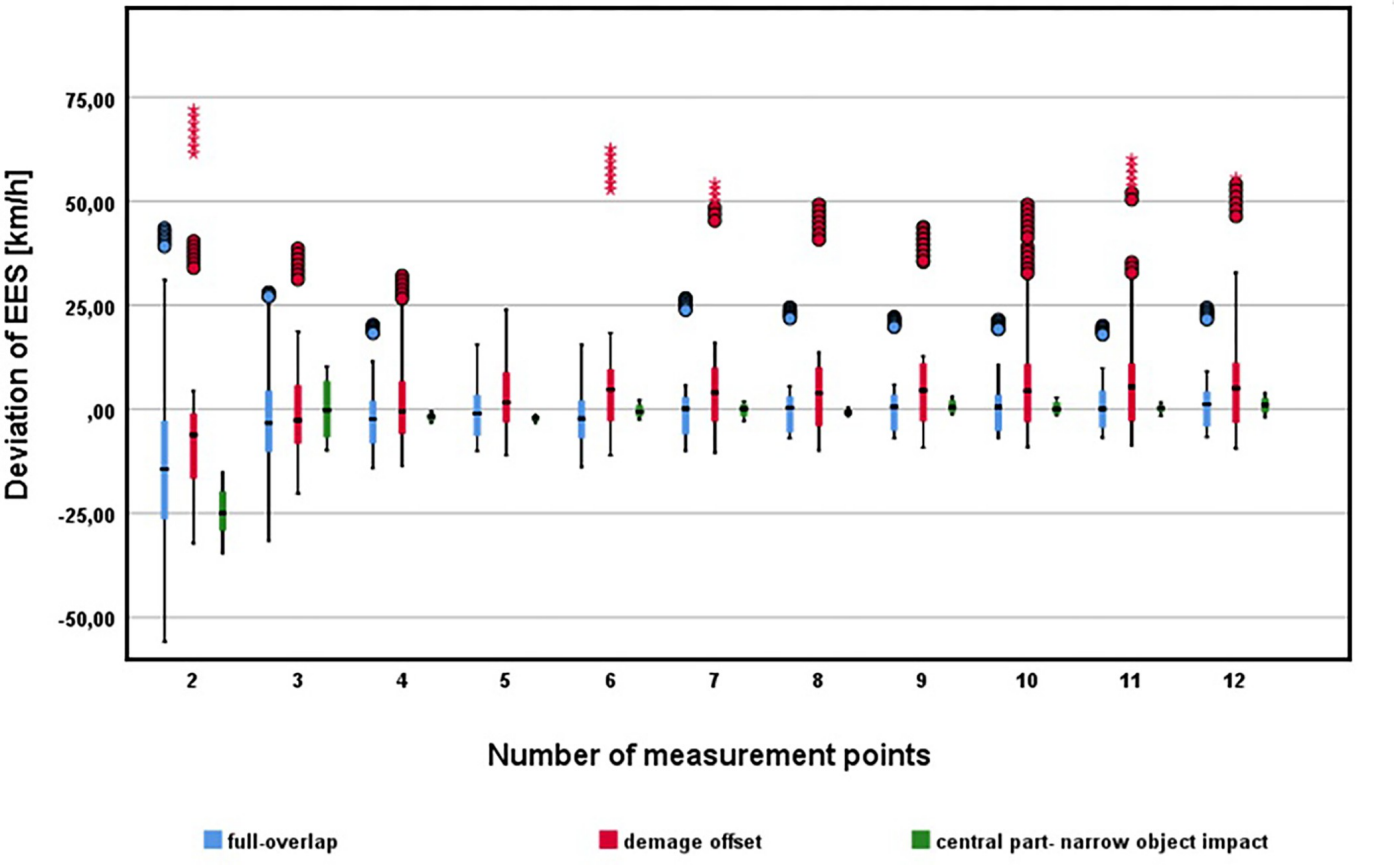
Effect of number of measurement points on the resulting EES with respect to extent of damage.

In comparison, using 2 measurement points which better reflect damage geometry, the EES calculation seems to be realistic and consistent across the entire dataset used (Fig 5).

### Limit speed b_0_

**Fig 6:**
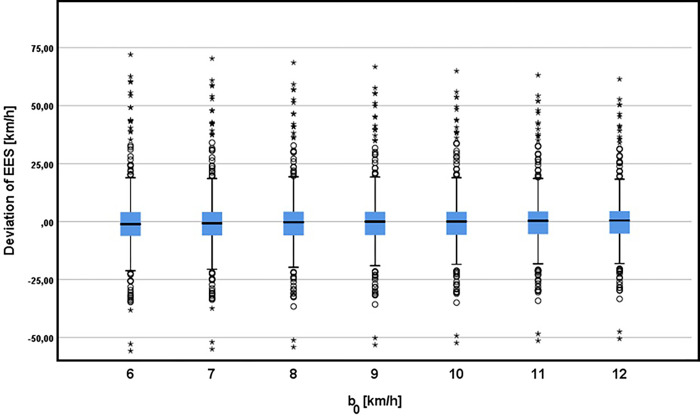
Effect of parameter b0 on the resulting EE.

ANOVA shows no statistically significant differences in the resulting EES values in relation to the used b0 values (Effect of parameter b0 on the resulting EES value illustrate Fig 6).

Vehicle category classification: within vehicle classes, b0 values have no statistically significant effect on the resulting EES values in case of small vehicles, as well as smaller medium-sized and medium-sized vehicles. Statistically significant differences were observed in case of SUVs, the results of groupwise comparison is shown in the following Table 2.

- Extent of damage and Overlap: ANOVA shows no statistically significant differences in the resulting EES values in relation to the used b_0_ values with respect to the extent of damage and overlap.

**Table 2:** Influence of b0 on resulting EES value–SUVs.

		Sig.
b_0_ = 6	b_0_ = 10	p < 0.05
	b_0_ = 11	p < 0.05
	b_0_ = 12	p < 0.01
b_0_ = 7	b_0_ = 11	p < 0.05
	b_0_ = 12	p < 0.01
b_0_ = 8	b_0_ = 12	p < 0.05
b_0_ = 9	b_0_ = 12	p < 0.05

### Deformation width

The groupwise comparison shows statistically significant differences between EES in relation to the used range of damage width (p-value less than 0.01)–bumper width or full vehicle width. Considering descriptive statistics (Table 3), the differences in the resulting EES values are very small. The observed effect size is not significant. The effect size is affected by the sample size.

**Fig 7:**
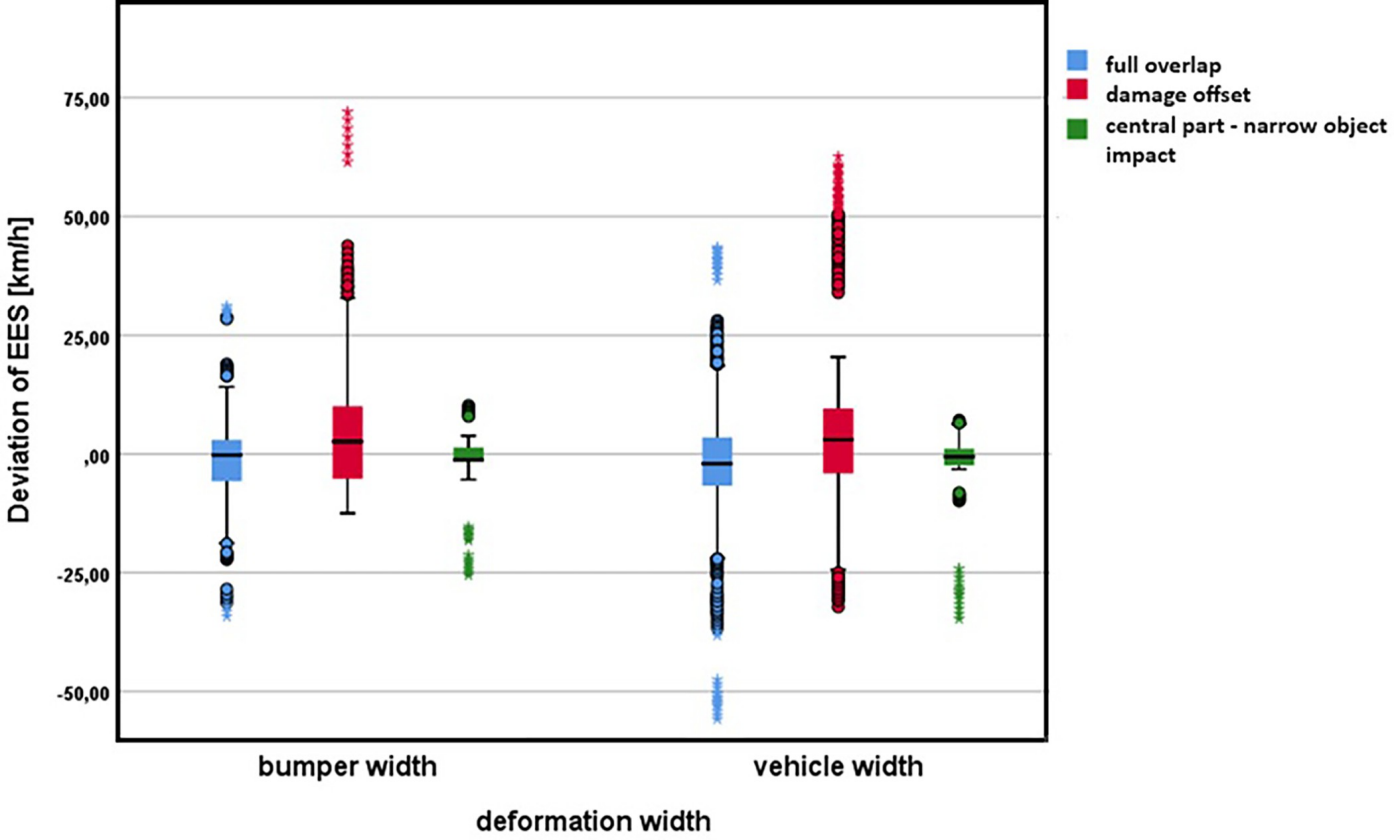
Effect of deformation width on the resulting EES with respect to damage extent.

- Vehicle category classification: within vehicle classes, there are statistically significant differences in the resulting EES value using different deformation width ranges (p-value less than 0.01) except for SUVs.
- Overlap: there are statistically significant differences in the resulting EES value using different deformation width ranges (p-value less than 0.05). The differences were significant in case of overlap up to 40% and higher than 60%.
- Extent of damage: considering extent of damage, there are statistically significant differences in the determined EES value with respect to the used methodology of deformation width in case of full overlap (p-value less than 0.01). For damage offset the effect was not significant (Effect of deformation width on the resulting EES with respect to damage extent illustrate Fig 7).

**Table 3:** Descriptive statistics–EES using different width values.

Width	EES [km/h]					
	mean	median	variance	std. deviation	minimum	maximum
**bumper**	43.960	41.802	296.583	17.222	5.800	134.000
**vehicle**	43.178	40.630	415.170	20.376	6.300	124.600

## Discussion

Determining EES using the CRASH3 calculation algorithm is influenced by numerous factors, especially stiffness, availability of investigated or comparable vehicles and the limited range of crash tests performed [18, 35]. Vangi [2] highlighted that the accuracy of the input data affects the resulting analysis of the crash, so the parameters entered into the EES calculation that could possibly affect the deformation energy calculation were selected for the purposes of this study. These variables were varied depending on selected factors such as the extent of damage, the type of collision (overlap) and also vehicle type (vehicle category classification). Even though some previous papers [11, 19, 20, 31] highlighted differences in quantified crush energy in relation to different methodologies and used variables range, most are not based on detailed analysis. The article aimed to analyse the parameters, such as the number of measurement points, the deformation width, and b_0_, on determining the deformation energy (specifically EES value) based on statistical analysis using the CRASH3 algorithm, as a suitable, efficient, and simple tool.

In terms of the number of measurement points, even considering the different characteristics of damage or vehicles (vehicle category classification, extent or nature of damage, overlap), there were significant differences in EES values especially if only 2 equally spaced measurement points were used. It can therefore be concluded that the use of 2 equally spaced measurement points should not be recommended in forensic practice. A similar conclusion follows from the calculation of the vehicle crush energy example described by Struble [26] but without detailed analysis. If using only 2 measurement points, instead of strictly equal spacing which is e.g. in case of full overlap measured at the edges of the vehicle body, the measurement should reflect the character and extent of the deformation. Significant distortion in the case of equal spacing could obviously be related mainly to the determination of the measurement points on the edge of the bodywork not considering rounded bodywork and hence measuring significantly low crush depth. Use of measurement points on the edges of impact bar leads to less distortion. The methodology described by Vangi (2020), which used approximation of the damaged area with triangular, rectangular or trapezoidal geometries, seems to provide a more realistic EES value even when using only 2 measurement points.

The determined EES value also differed significantly in the whole dataset using less than 6 measurement points, so for practical use, it seems appropriate to use at least 6 measurement points, as is widely used in forensic practise [2, 20, 26, 28, 30, 31]. Even in the case of 5 or 6 measurement points, the results are statistically significantly different from the EES values obtained using more measurement points, even though the differences in calculated EES and measured EES are not high, especially with respect to the inaccuracy/technically acceptable tolerance of the EES determination. These results confirmed the conclusions highlighted by Struble [25], that even though 6 points are widely used, 7 well-chosen points appear to suffice, even for a severe side impact. As can be seen in the conducted statistical analysis, a higher number of measurement points in case of equally spaced measurements leads to a more accurate EES calculation, because it usually better reflects the actual shape of the vehicle damage. Using more than 7 measurement points does not differ significantly in comparison to 7 measurement points, which confirmed its usability in forensic practise.

Equal spacing is not generally required but it could simplify the calculation process, so is recommended by some calculation software. Based on the CRASH3 technical manual, equal spacing was recommended, so it was analysed in this paper. Placement of measurement points should respect location of impact bar.

Use of the CRASH3 algorithm assumes that the vehicle damage provides a suitable basis for assessing the energy dissipated and the contacting surface reaches a common velocity with the struck object, so the algorithm is not suitable for analysis of under-runs, sideswipes, highly offset or highly oblique impacts [16]. Used crashes with 25–30% overlap due to higher impact speed resulted in larger scale damage, so the CRASH3 algorithm could be appropriately used for analysis, but the obtained results do not reflect the small overlap collisions and should undergo further analysis.

A more detailed analysis of different measurement point numbers shows that collisions with narrow obstacle that caused extensive deformation, especially in the area of the vehicle center, are problematic when quantifying the damage. During a collision with a narrow obstacle, longitudinals, which should primarily absorb the energy during the collision, are not hit, as stated by Struble [31] The results confirmed that the number of measurement points should be chosen with respect to the deformation characteristics as described by, e.g. Nordhagen et al. [27], but the use of measurement points could not be evaluated validly since the CRASH3 algorithm is generally less suitable for calculating EES after a collision with a narrow obstacle [16, 18, 36, 37].

Among the discussed parameters belongs b_0_, which represents the impact speed at which no residual structural damage is expected to occur. In our analysis the used range (6–12 km/h) of b_0_ values chosen based on literature review [2, 11, 14, 23–25] did not lead to statistically significant differences in the resulting EES in the whole dataset. The used b_0_ range had a significant effect on the resulting EES value only in the case of SUVs. These vehicles show greater stiffness, which supposes the use of lower b_0_ values should not be recommended. So even though the variation of the b_0_ value did not result in significant differences in the EES value, use of the b_0_ value should respect the vehicle stiffness considering class, technological obsolescence, or degree of corrosion. These factors were defined as influencing vehicle stiffness including b_0_ also by [2, 20, 26, 27, 33], but not based on statistical analysis.

Although the used range of deformation width proved to be a significant variable, the observed effect size is not significant in the sense of practical use. The analysed sample includes various vehicle classes with differing extent of damage. The dataset was therefore tested with respect to these different characteristics. It is necessary to conclude that deformation width is one of the variables which should be carefully measured for the purpose of calculating EES value.

The paper faced several limitations. One of the limitations could be seen in the sample size, which consists of 20 vehicles with differing stiffness characteristics and damage but low variability in the age of the vehicles, which could affect the obtained results. The result could have been biased by an insufficient dataset that did not reflect technological obsolescence or corrosion. Increasing the sample size with respect to the different types of crashes (especially increasing the number of narrow object impacts, crashes with SUVs at higher impact speeds, small overlap crashes, etc.) could also help to eliminate distortion in the results. Placement of measurement points should respect location of impact bar. It is also necessary to consider the limitations of the CRASH3 algorithm (such as the mentioned overestimation in case of narrow obstacle collisions or small overlap collisions).

The presented paper is focused on using the CRASH3 algorithm, so the methodology used is based on the CRASH3 manual [34] which recommend equal spacing. Tumbas and Smith [28] stated that maximum deformation depth in the sector should replace one of the equidistant measured values (if maximum crush is not recorded from one of the equidistant stations) to better reflect the deformation profile. Due to the widely used equally spaced measurement point procedure (which simplifies the calculation process), the article was focused on the analysis of equal spacing. Uneven distribution of measurement points should be the subject of further studies.

For the purpose of calculating EES in this paper, the NHTSA database was used to find the necessary vehicle substitutes. The CRASH3 algorithm’s accuracy is mainly influenced by the accuracy of the stiffness coefficient [11]. American vehicles may differ structurally in comparison to European vehicles, so may differ in stiffness even in the same vehicle [18, 38, 39]. This paper did not focus on a selection of these parameters even though the choice of vehicle substitutes also affects the EES calculation.

## Conclusion

The accuracy of crash analysis, which can significantly influence determination of culpability of the crash, is directly influenced by the accuracy of the input parameters, thus is highly dependent on the accuracy of the crash documentation (including vehicle deformation). Time constraints for data collection at individual crash sites, associated with, e.g. traffic congestion, can result in less accurate data or even the absence of some essential data.

To determine the vehicle impact speed it is necessary to quantify the deformation energy. Deformation energy can be expressed by Equivalent Energy Speed (EES). The main contribution of this article was the analysis of selected input variables necessary for calculating EES including the deformation depth and deformation width variables, which can be directly influenced by the quality of the documentation on the crash site. The results confirmed that the range of used variables needs to be selected with respect to the deformation character and vehicle type. The results serve as practical recommendations for refining and therefore improving the quality of crash analysis. As part of subsequent research activities, the influence of deviations in the measurement of input values by selected methods on the resulting determination of the vehicle’s impact speed should be quantified.

## Abbreviations

CRASH3: Calspan Reconstruction of Accident Speeds on the Highway, version 3
EES: Equivalent Energy Speed [km/h]
LUV: Light utility vehicle
SUV: Sport utility vehicle
